# Cost impact of procalcitonin-guided decision making on duration of antibiotic therapy for suspected early-onset sepsis in neonates

**DOI:** 10.1186/s13054-021-03789-x

**Published:** 2021-10-20

**Authors:** A. J. L. M. Geraerds, Wendy van Herk, Martin Stocker, Salhab el Helou, Sourabh Dutta, Matteo S. Fontana, Frank A. B. A. Schuerman, Rita K. van den Tooren-de Groot, Jantien Wieringa, Jan Janota, Laura H. van der Meer-Kappelle, Rob Moonen, Sintha D. Sie, Esther de Vries, Albertine E. Donker, Urs Zimmerman, Luregn J. Schlapbach, Amerik C. de Mol, Angelique Hoffman-Haringsma, Madan Roy, Maren Tomaske, René F. Kornelisse, Juliette van Gijsel, Eline G. Visser, Annemarie M. C. van Rossum, Suzanne Polinder

**Affiliations:** 1 Department of Public Health, Erasmus MC, University Medical Center Rotterdam, P.O. Box 2040, 3000 CA Rotterdam, The Netherlands; 2 Division of Paediatric Infectious Diseases & Immunology, Department of Paediatrics, Erasmus MC University Medical Centre - Sophia Children’s Hospital, Rotterdam, The Netherlands; 3 Department of Paediatrics, Neonatal and Paediatric Intensive Care Unit, Children’s Hospital Lucerne, Lucerne, Switzerland; 4 Division of Neonatology, McMaster University Children’s Hospital, Hamilton Health Sciences, Hamilton, ON Canada; 5 Neonatal Intensive Care Unit, Isala Women and Children’s Centre, Isala Hospital, Zwolle, The Netherlands; 6 Department of Paediatrics, Haaglanden Medical Center, ‘s Gravenhage, The Netherlands; 7 Neonatal Unit, Department of Obstetrics and Gynaecology, Motol University Hospital, Second Medical Faculty, Charles University, Prague, Czech Republic; 8 Institute of Pathological Physiology, First Medical Faculty, Charles University, Prague, Czech Republic; 9 Department of Neonatology, Reinier de Graaf Gasthuis, Delft, The Netherlands; 10 Department of Neonatology, Zuyderland Medical Centre, Heerlen, The Netherlands; 11 Department of Neonatology, Amsterdam UMC, Vrije Universiteit Amsterdam, Amsterdam, The Netherlands; 12 Department of Paediatrics, Jeroen Bosch Hospital, ‘s-Hertogenbosch, The Netherlands; 13 Department of Paediatrics, Maxima Medical Centre, Veldhoven, The Netherlands; 14 Department of Paediatrics, Kantonsspital Winterthur, Winterthur, Switzerland; 15 Department of Paediatrics, Bern University Hospital, Inselspital, University of Bern, Bern, Switzerland; 16 Paediatric Critical Care Research Group, Mater Research Institute, University of Queensland, Brisbane, QLD Australia; 17 Paediatric Intensive Care Unit, Lady Cilento Children’s Hospital, Brisbane, QLD Australia; 18 Department of Neonatology, Albert Schweitzer Hospital, Dordrecht, The Netherlands; 19 Department of Neonatology, Sint Franciscus Gasthuis, Rotterdam, The Netherlands; 20 Department of Neonatology, St. Josephs Healthcare, Hamilton Health Sciences, Hamilton, ON Canada; 21 Department of Paediatrics, Stadtspital Triemli, Zürich, Switzerland; 22 Division of Neonatology, Erasmus MC University Medical Centre-Sophia Children’s Hospital, Rotterdam, The Netherlands; 23 Julius Training General Practitioner, University Medical Centre Utrecht, Utrecht, The Netherlands

**Keywords:** Neonates, Procalcitonin-guided decision making, Sepsis, Costs

## Abstract

**Backgrounds:**

The large, international, randomized controlled NeoPInS trial showed that procalcitonin (PCT)-guided decision making was superior to standard care in reducing the duration of antibiotic therapy and hospitalization in neonates suspected of early-onset sepsis (EOS), without increased adverse events. This study aimed to perform a cost-minimization study of the NeoPInS trial, comparing health care costs of standard care and PCT-guided decision making based on the NeoPInS algorithm, and to analyze subgroups based on country, risk category and gestational age.

**Methods:**

Data from the NeoPInS trial in neonates born after 34 weeks of gestational age with suspected EOS in the first 72 h of life requiring antibiotic therapy were used. We performed a cost-minimization study of health care costs, comparing standard care to PCT-guided decision making.

**Results:**

In total, 1489 neonates were included in the study, of which 754 were treated according to PCT-guided decision making and 735 received standard care. Mean health care costs of PCT-guided decision making were not significantly different from costs of standard care (€3649 vs. €3616). Considering subgroups, we found a significant reduction in health care costs of PCT-guided decision making for risk category ‘infection unlikely’ and for gestational age ≥ 37 weeks in the Netherlands, Switzerland and the Czech Republic, and for gestational age < 37 weeks in the Czech Republic.

**Conclusions:**

Health care costs of PCT-guided decision making of term and late-preterm neonates with suspected EOS are not significantly different from costs of standard care. Significant cost reduction was found for risk category ‘infection unlikely,’ and is affected by both the price of PCT-testing and (prolonged) hospitalization due to SAEs.

**Supplementary Information:**

The online version contains supplementary material available at 10.1186/s13054-021-03789-x.

## Background

Early-onset sepsis (EOS) is one of the main causes for hospitalization in the first week of life. Annually, approximately 4–7% of term and late-preterm neonates in high-income countries are treated with intravenous antibiotics because of suspected EOS, whereas the prevalence of EOS is only 0.1% [1]. This implicates unnecessary antibiotic treatment in the majority of the treated neonates. Moreover, antibiotic treatment is associated with undesirable consequences, such as hospital admission, neonatal and parental discomfort, alterations in the neonatal microbiome and the use of health care resources, which puts a high demand on health care costs [2, 3].

One approach to improve the management of EOS is by shortening the duration of antibiotic treatment, using biomarker guidance. A successful biomarker-based strategy that was identified in previous studies is procalcitonin (PCT)-guided decision making [4–6]. In the NeoPInS study, PCT-guided decision making was found to significantly reduce the duration of antibiotic treatment, with unchanged outcome for adverse events [7]. Whether PCT-guided decision making is also cost-effective has not been evaluated yet. The main reason for concerns with respect to cost-effectiveness is the price of a PCT-test: a PCT-test is more expensive than a CRP-test. A previous patent on the PCT-test has expired, which has led to more market competition, and therefore a lower price for the PCT-test kit. This has resulted in lower costs of PCT-testing and might therefore result in PCT-guided decision making being more cost-effective.

There have been cost analyses of PCT-guided decision making in critically ill adult patients [8–10], and in patients with sepsis and lower respiratory tract infection (LRTI) [11, 12]. Voermans et al. and Mewes et al. found that PCT-guided decision making led to a cost saving in both sepsis patients and LRTI patients, mainly due to a reduced duration of hospitalization [12], but also due to a reduction in antibiotic days, shorter duration of mechanical ventilation and fewer patients at risk for antibiotic resistant or C. difficile infection [11]. For critically ill patients, it was found that PCT-guided decision making led to significantly lower hospital costs and shorter duration of hospitalization [8, 9]. However, Kip et al. found no significant difference in total health care-related costs [10].

To our knowledge, no study has investigated health care use and costs of PCT-guided decision making in children. This paper, based on the NeoPInS study, describes whether PCT-guided decision making for neonates with suspected EOS can safely reduce medical costs. In addition, it explores differences in costs between subgroups based on country, risk classification and gestational age.

## Methods

### Study design

This study explored the total direct medical costs of PCT-guided decision making in neonates with suspected EOS from a hospital-based perspective. Assessment was based on the Neonatal PCT Intervention Study (NeoPInS), a randomized open controlled international multicenter intervention trial. Patients were enrolled in 18 hospitals, situated in the Netherlands (*n* = 11), Switzerland (*n* = 4), Canada (*n* = 2) and the Czech Republic (*n* = 1). Ethical approval of the protocol was obtained. Furthermore, written informed consent was obtained for all study participants. For more detailed information regarding the methods, study design and participants of the NeoPInS study, we refer to the original paper [7, 13]. The most important aspects are presented below.

### Participants

Neonates born between May 21, 2009 and February 14, 2015 after 34 weeks of gestational age with suspected EOS in the first 72 h of life requiring antibiotic therapy were eligible for inclusion. This study only included neonates with no missing key variables. Therefore, we excluded 4 neonates from the PCT-guided decision-making group, and 17 neonates from the standard care group that were specified in the original paper [7].

### Procedures

Within 12 h after start of therapy, all neonates were stratified into four risk categories, based on three elements: clinical symptoms, risk factors and conventional laboratory measurements (Additional file 1: Figure S1). The four categories were: category 1 (neonates with a *proven* infection with a positive blood culture), category 2 (neonates with *high risk* of infection due to clinical symptoms, risk factors and abnormal laboratory findings), category 3 (neonates with *a possible* risk of infection based on two out of three elements of the risk stratification) and category 4 (neonates where infection is *unlikely*, based on one out of three elements from the risk stratification). All neonates in categories 1 and 2 were given standard care according to local policy (a minimum of 7 days of antibiotic treatment). PCT-guided decision making was not applied since their high risk of infection would not allow shorter antibiotic treatment. Therefore, neonates in category 1 and 2 were excluded in this study. The antibiotic treatment of neonates in category 3 and 4 was based on either PCT-guided decision making (intervention) or standard care (based on local policy) (Additional file 1: Figure S1).

PCT-guided decision making was defined by a minimum treatment duration of 24 h, where the decision to discontinue antibiotic treatment was based on the measured procalcitonin values: antibiotic therapy could be discontinued when two consecutive PCT values were within normal values according to the postpartum nomogram [7]. Physicians decided when a neonate was discharged from the hospital, and were at all times allowed to overrule the recommendation from the treatment-related algorithm based on for example clinical symptoms or other laboratory investigations.

### Resource data

During the trial, data were retrieved from individual patient records in a case record form (CRF) by local investigators. A monitoring team performed 100% source data verification through onsite visits, in order to ensure data quality and completeness. However, due to unavailability of key information in some patient files, some missing values in key variables remained. Database access was restricted to the data management team until the end of the trial.

For all patients, follow-up information on the first month of life was obtained, regarding recurrence of infection, readmission to the hospital, additional courses of antibiotics and death. Information on serious adverse events (SAEs) (e.g., hyperbilirubinemia, feeding problems due to prematurity) was retrieved by monitoring.

### Cost study

As no effect of PCT-guided decision making compared to standard care was found in mortality and morbidity [7], a cost minimization analysis was conducted. The cost minimization analysis comprised a direct comparison between total health care costs of PCT-guided decision making and standard care, from a health care perspective. All health care resources that were used in the hospital were registered in a CRF. Additional days of hospitalization and readmission due to SAEs were included in the total duration of hospitalization, and additional antibiotic treatment was included in the total duration of antibiotics treatment. Estimates of unit costs were based on the Dutch guideline prices, or were obtained from the website of the Dutch Health Insurance Board [14, 15]. The cost price of PCT-testing was calculated based on the micro-costing method. The mean of all prices of PCT-testing, which were retrieved from the participating hospitals, was used as the price per PCT-test. Total medical costs were calculated by multiplying volumes of health care resources with corresponding unit prices, and consisted of costs of hospital days, costs of antibiotics, costs of laboratory tests (CRP and PCT) and labor costs. Costs were calculated in the European currency (Euro), corrected for inflation for the year 2015. All prices included in this cost study are presented in Additional file 2 (Table S1). Discounting was not applied, since the follow-up period of the study comprised one month.

Unit prices for Switzerland (CH), Canada (CA) and the Czech Republic (CZ) were based on the Dutch prices, corrected for the purchasing power parity (PPP) for the general domestic product (GDP). Subgroups based on country, age (gestational age < 37 weeks or ≥ 37 weeks) and risk category were compared to explore potential cost drivers.

### Sensitivity analysis

Sensitivity analysis was performed by varying costs of PCT-testing, to study the influence of changes in pricing on the results. Costs of PCT-testing were examined using the minimum (NL €13.70; CH €17.50; CA €13.50; CZ €7.80) and maximum prices (NL €33.90; CH €43.40; CA €33.40; CZ €19.50). A second sensitivity analysis was performed by excluding additional days of hospitalization due to SAEs (e.g., hyperbilirubinemia/feeding problems), with the aim to provide insight in the effect of the SAEs on the total health care costs. The SAEs were deemed by the data safety and monitoring board as not-related to the study. A third sensitivity analysis was performed using the per-protocol population, in order to express the real potential of PCT-guided decision making. Neonates with protocol violations were excluded in the per-protocol analyses.

### Statistical analysis

An intention-to-treat analysis was performed to express the real clinical situation of PCT-guided decision making. For clinical and demographical data, a comparison between groups for categorical variables was made using either the *χ*^2^ test or the Fisher exact test when appropriate. For continuous variables, nonparametric analysis was performed using the Mann–Whitney U test. *P* values < 0.05 were considered to indicate statistical significance. Variables with multiple categories, such as country and delivery method, were compared using the Kruskal–Wallis test.

Mean costs were reported in euro (€) with the interquartile range (IQR). Differences in costs between the PCT-guided decision-making group and the standard care group were compared using Mann–Whitney U test, since costs were not normally distributed. Due to the exploratory nature of the subgroup analyses, no correction for multiplicity was applied. Analyses were performed using SPSS software package, version 25 (SPSS Inc., Chicago, Illinois, USA).

## Results

### Study population

**Fig. 1:**
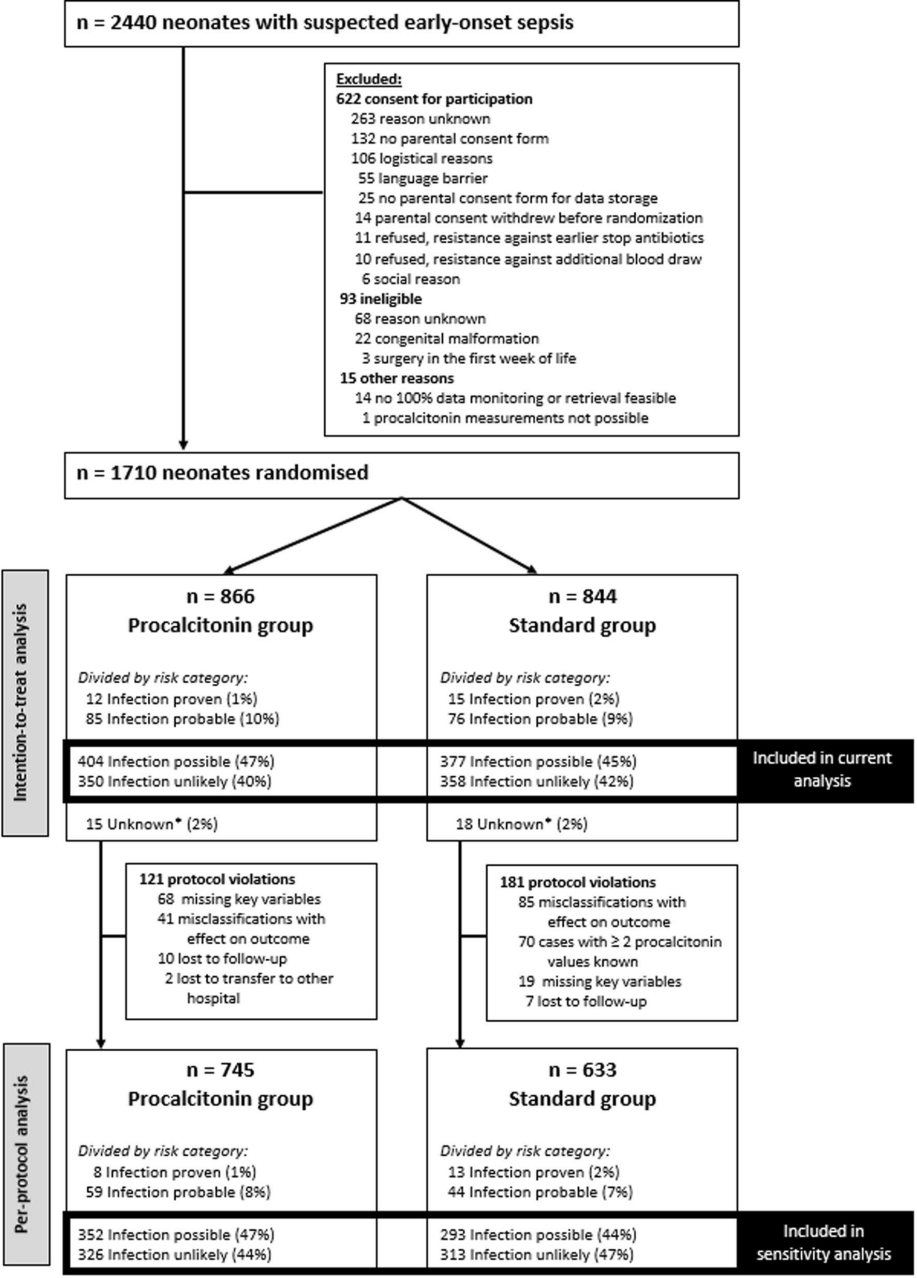
Flowchart. *Unknown are neonates with missing key variables

Overall, 2440 neonates with suspected EOS were screened, of which 730 neonates were excluded for reasons described in the original study [7]. In this study, we excluded 21 more neonates due to missing information on duration of hospitalization and antibiotic treatment. Of the remaining 1689 neonates, 1489 were classified as risk category ‘infection possible’ or ‘infection unlikely, and randomly assigned to either PCT-guided decision making (*n* = 754) or standard care (*n* = 735) (Fig. 1). No significant differences in baseline characteristics were found between the PCT-guided decision-making group and the standard care group (Table 1). For the sensitivity analyses in the per protocol population, neonates with protocol violations were excluded. This resulted in the inclusion of 678 neonates in the per protocol analysis of the PCT-guided decision-making group, and 606 neonates in the standard care group.

**Table 1:** Patient characteristics of intention to treat population

	PCT-guided decision making *n* = 754	Standard care *n* = 735	*P* value
Male sex	452 (59.9%)	428 (58.2%)	0.501
Country			0.440
Netherlands	439 (58.2%)	442 (60.1%)	
Switzerland	124 (16.4%)	111 (15.1%)	
Canada	152 (20.2%)	155 (21.1%)	
Czech Republic	39 (5.2%)	27 (3.7%)	
Gestational age (in weeks)	38.5 (2.2)	38.5 (2.2)	0.731
Birth weight in kg	3.3 (0.66)	3.4 (0.62)	0.346
Delivery way			0.051
Spontaneous vaginal delivery	375 (49.7%)	331 (45.0%)	
Vacuum/forceps delivery	114 (15.1%)	133 (18.1%)	
C-section	263 (34.9%)	270 (36.7%)	
*Missing*	*2 (0.3%)*	*1 (0.1%)*	
Arterial cord pH	7.2 (0.1)	7.2 (0.1)	0.371
*Missing*	*135 (17.9%)*	*142 (19.3%)*	
Apgar score			
1 min postpartum	7.4 (2.2)	7.3 (2.3)	0.378
*Missing*	*1 (0.1%)*	*6 (0.8%)*	
5 min postpartum	8.6 (1.6)	8.5 (1.7)	0.245
*Missing*	*0 (-)*	*8 (1.1%)*	
10 min postpartum	8.9 (1.3)	8.9 (1.3)	0.567
*Missing*	*276 (36.6%)*	*255 (34.7%)*	
Risk category			0.377
Infection possible (medium risk)	404 (53.6%)	377 (51.3%)	
Infection unlikely (low risk)	350 (46.4%)	358 (48.7%)	

The italics were used to represent missing values per variable

Data are expressed as mean (SD) or *n* (%)

*SD* standard deviation

^*^Significant at a 5% level (*P* < 0.05)

### Duration of hospitalization and antibiotic treatment

In all countries combined and individually, neonates treated with PCT-guided decision making had on average a shorter duration of antibiotics treatment than neonates treated following standard care (Table 2). However, mean duration of hospitalization differed between countries, with slightly shorter hospital stay for the standard care group in all countries combined (2 h), mainly caused by a shorter duration of hospitalization in the Czech Republic (96 h). The Netherlands, Switzerland and Canada, on the other hand, had on average a shorter duration of hospitalization for the PCT-guided decision-making group than for the standard care group (minus 1, 16 and 5 h respectively). Comparing countries, it was found that Switzerland had on average the longest duration of hospitalization, both for the PCT-guided decision-making group and for the standard care group. The Netherlands had on average the shortest duration of hospitalization for the PCT-guided decision-making group, whereas the Czech Republic had the shortest duration of hospitalization for standard care. For both PCT-guided decision making and standard care, duration of antibiotics treatment was on average shortest in Canada. Longest duration of antibiotic treatment was found for the Czech Republic (PCT-guided decision making) and Switzerland (standard care).

**Table 2:** Mean [IQR] duration of hospitalization and antibiotic treatment in hours per country

	PCT-guided decision making (*n* = 754)		Standard care (*n* = 735)	
	Duration of hospitalization mean [IQR]	Duration of antibiotic treatment mean [IQR]	Duration of hospitalization mean [IQR]	Duration of antibiotic treatment mean [IQR]
All countries	164 [69, 184]	74 [32, 114]	162 [79, 183]	87 [49, 120]
The Netherlands	134 [63, 165]	72 [30, 120]	135 [71, 166]	88 [51, 130]
Switzerland	235 [120, 303]	86 [42, 120]	251 [143, 289]	101 [60, 121]
Canada	181 [81, 192]	67 [30, 78]	186 [87, 218]	74 [47, 71]
Czech Republic	207 [116, 288]	91 [36, 133]	111 [69, 146]	96 [58, 132]

Data are expressed in hours as mean + IQR (interquartile range)

Antibiotic treatment includes both IV and oral treatment

### Costs

Overall, it was found that costs of PCT-guided decision making were higher than costs of standard care, but not significantly (€3649 vs. €3616, *P* = 0.240) (Table 3). Subgroup analysis showed that costs were significantly different between countries. However, costs were not significantly different between PCT-guided decision making and standard care within all countries, except for the Czech Republic, where standard care had significantly lower costs than PCT-guided decision making (€1242 versus €2328, *P* < 0.001).

**Table 3:** Costs of health care use per patient for PCT-guided decision making and standard care (2015€)

	PCT-guided decision making (*n* = 754)						Standard care (*n* = 735)					
	All countries	The Netherlands	Switzerland	Canada	Czech Republic	*P* value^a^	All countries	The Netherlands	Switzerland	Canada	Czech Republic	*P* value^a^
All patients	3649 [1496, 4091]	2750 [1315, 3304]	5813 [3073, 7415]	4823 [2271, 5062]	2328 [1360, 3160]*	< 0.001*	3616 [1639, 4372]	2710 [1456, 3272]	6091 [3579, 6965]	4844 [2366, 5867]	1242 [780, 1618]*	< 0.001*
*Per risk category*												
Infection possible (medium risk)	4247 [1989, 4833]	3375 [1667, 3726]	6324 [3376, 7957]	4511 [2221, 4850]	2294 [1240, 3337]*	< 0.001*	4238 [2023, 5021]	3260 [1863, 3467]	6474 [3350, 7947]	4867 [2298, 6343]	1279 [812, 1768]*	< 0.001*
Infection unlikely (low risk)	2960 [1295, 3389]*	2255 [1210, 3062]*	3977 [2404, 5346]*	5218 [2277, 5526]	2415 [1730, 3160]*	< 0.001*	2962 [1424, 3591]*	2267 [1316, 3166]*	4958 [3781, 6314]*	4818 [2495, 5046]	1195 [628, 1559]*	< 0.001*
*P* value^a^	< 0.001*	< 0.001*	0.004*	0.864	0.508		< 0.001*	< 0.001*	0.322	0.697	0.456	
*Per gestational age (GA)*												
GA < 37 weeks	6436 [2853, 8728]	5609 [2638, 8653]	9760 [6407, 11596]	5466 [2302, 5610]	2978 [1500, 4267]*	< 0.001*	6098 [2881, 7945]	5354 [2779, 7349]	9045 [4858, 11572]	4867 [2648, 6580]	884 [605, 1263]*	< 0.001*
GA ≥ 37 weeks	2928 [1376, 3468]	2234 [1276, 3150]*	4069 [2615, 4794]*	4585 [2173, 4989]	2133 [1174, 2913]*	< 0.001*	2986 [1538, 3494]	2245 [1416, 3141]*	4427 [3253, 5127]*	4836 [2315, 5681]	1304 [812, 1768]*	< 0.001*
*P* value^a^	< 0.001*	< 0.001*	< 0.001*	0.454	0.269		< 0.001*	< 0.001*	< 0.001*	0.524	0.216	

Data are expressed as mean + IQR (interquartile range)

Costs are expressed in € corrected for inflation for the year 2015

^*^Significant at a 5% level (*P* < 0.05)

^a^*P* value for comparison between the subgroups (country, risk category or gestational age)

If costs are ended with *, this indicates statistically significant difference between the PCT-guided decision-making group and standard care group

**Fig. 2:**
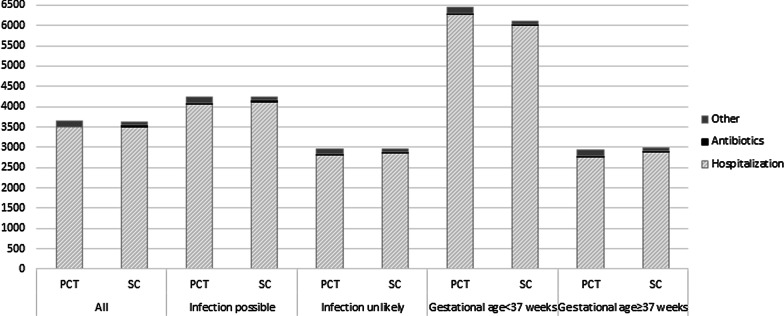
Composition of total health care costs for PCT-guided decision making and standard care for subgroups. *PCT* Procalcitonin-guided decision-making group. *SC* Standard care group. *Other includes costs of laboratory tests and costs of labor

Considering all countries together, subgroup analysis of costs per risk category showed on average significantly lower costs for PCT-guided decision making compared to standard care in risk category ‘infection unlikely’ (*P* = 0.041) (Fig. 2). Costs for risk category ‘infection possible’ were found to be slightly higher for the PCT-guided decision-making group, but not significantly. Considering risk categories within countries, it was found that in both the Netherlands, Switzerland and the Czech Republic costs were significantly lower for PCT-guided decision making in risk category ‘infection unlikely.’ In addition, for the Czech Republic significantly higher costs of PCT-guided decision making were found for risk group ‘infection possible.’ No significant differences between PCT-guided decision making and standard care were found for Canada.

Late preterm neonates (gestational age < 37 weeks) had significantly higher costs compared to term neonates (gestational age ≥ 37 weeks), both for PCT-guided decision making (€6436 vs. €3081, *P* < 0.001) and for standard care (€6098 vs. €2986, *P* < 0.001), considering all countries together. Similar significant results were found for the Netherlands and Switzerland, whereas for Canada and the Czech Republic the difference was not significant. Subgroup analyses of gestational age groups within countries showed a significant difference between PCT-guided decision making and standard care for Switzerland in gestational age ≥ 37 weeks group only, and for the Czech Republic in both gestational age < 37 weeks and ≥ 37 weeks.

Considering total health care use, it was found that all health care use, except for hospital hours, was significantly different (*P* < 0.05) between the two treatment groups (Table 4). The main contributor to health care costs was costs of hospitalization, both for PCT-guided decision making and standard care (Fig. 2).

**Table 4:** Resource use and costs of health care (2015€) per patient for the population

	PCT-guided decision making (*n* = 754)		Standard care (*n* = 735)		*P* value^a^
	Resource use (number)	Costs (euro)	Resource use (number)	Costs (euro)	
PCT number of tests	3 [2, 4]	63 [39, 79]	NA	NA	< 0.001*
CRP number of tests	4 [3, 4]	15 [12, 16]	3 [3, 4]	14 [10, 16]	0.001*
Hospital hours	164 [69, 184]	3489 [1377, 3906]	162 [79, 183]	3511 [1541, 4215]	0.060
Antibiotic doses	10 [5, 12]	22 [9, 25]	12 [7, 14]	26 [13, 29]	< 0.001*
Labor costs	–	61 [43, 76]	–	66 [52, 79]	< 0.001*
*Total costs*	–	3649 [1496, 4091]	–	3616 [1639, 4372]	0.240

Data are expressed as mean + IQR (interquartile range)

Costs are expressed in € corrected for inflation for the year 2015

^a^*P* value for the difference in costs between PCT-guided decision making and standard care

^*^Significant at 5% level (*P* < 0.05)

NA = not applicable

### Sensitivity analysis

Differences in mean health care costs between the PCT-guided decision-making group and the standard care group remained insignificant, using both the minimum and maximum price of a PCT-test (Table 5). However, for risk category ‘infection unlikely,’ it was found that the difference in costs between the two groups was no longer significant when using the maximum price for a PCT-test, whereas it was significant when using the minimum price. For the Netherlands, it was found that the difference in costs was significant when using the minimum price for PCT-testing, whereas it was no longer significant when the maximum price for PCT-testing was used. For the Czech Republic, the difference in costs remained significant using both the minimum and maximum price.

**Table 5:** Costs of care with lowest and highest price for PCT-test (2015€)

	PCT-guided decision making (*n* = 754)		Standard care (*n* = 735)		*P* value	
	Min. price	Max. price	Min. price	Max. price	Min. price	Max. price
All patients (in total)	3630 [1479, 4073]	3695 [1533, 4137]	3616 [1639, 4372]	3616 [1639, 4372]	0.154	0.534
*Per risk category*						
Infection possible (medium risk)	4226 [1964, 4798]	4296 [2033, 4916]	4238 [2023, 5021]	4238 [2023, 5021]	0.648	0.938
Infection unlikely (low risk)	2942 [1281, 3370]	3001 [1330, 3441]	2962 [1424, 3591]	2962 [1424, 3591]	0.023*	0.112
*Per country*						
Netherlands	2731 [1296, 3292]	2793 [1352, 3372]	2710 [1456, 3272]	2710 [1456, 3272]	0.048*	0.288
Switzerland	5783 [3044, 7388]	5884 [3137, 7485]	6091 [3579, 6965]	6091 [3579, 6965]	0.190	0.330
Canada	4809 [2259, 5051]	4855 [2299, 5119]	4844 [2366, 5867]	4844 [2366, 5867]	0.102	0.151
Czech Republic	2312 [1343, 3146]	2366 [1401, 3193]	1242 [780, 1618]	1242 [780, 1618]	< 0.001*	< 0.001*

Data are expressed as mean + IQR (interquartile range).

*Min* minimum, *Max* maximum, *IQR* interquartile range

*Significant at 5% level (*P* < 0.05)

Costs are expressed in € corrected for inflation for the year 2015

The sensitivity analyses excluding additional days of hospitalization due to SAEs showed that mean duration of hospitalization was significantly (*P* < 0.001) shorter for PCT-guided decision making than for standard care (Table 6). In addition, mean total health care costs were significantly (*P* < 0.001) lower for PCT-guided decision making compared to standard care.

**Table 6:** Mean duration of hospitalization and costs of care excluding duration of hospitalization due to SAEs, and excluding neonates with protocol violations

	PCT-guided decision making	Standard care	*P* value
*Excluding SAEs*			
Duration of hospitalization in hours	97 [48, 139]	107 [64, 146]	< 0.001*
Total costs of health care in euro	2166 [1132, 3084]	2349 [1387, 3087]	< 0.001*
*Excluding neonates with protocol violations*			
Duration of hospitalization in hours	162 [67, 181]	159 [77, 181]	0.035*
Total costs of health care in euro	3735 [1496, 4159]	3578 [1636, 4293]	0.412

Data are expressed as mean + IQR (interquartile range)

*SAE* Serious adverse event

*Significant at 5% level (*P* < 0.05)

Costs are expressed in € corrected for inflation for the year 2015

Sensitivity analyses of the per protocol population, comparing duration of hospitalization including hospitalization due to SAEs and total costs of health care including SAEs, showed a significant difference in duration of hospitalization between PCT-guided decision making and standard care (162 h versus 159 h, *P* = 0.035). However, total costs of health care were not significantly different between PCT-guided decision making and standard care.

## Discussion

### Main findings

This study compared medical costs of neonates born after 34 weeks of gestational age, who had suspected low risk of EOS in the first 72 h of life and required antibiotic therapy, between an intervention (PCT-guided decision-making) and control group (standard care according to local policy). Neonates were considered to have a low risk when they were classified in either risk category ‘infection possible’ or ‘infection unlikely.’ Mean total costs of health care were not significantly different between the PCT-guided decision-making group and the standard care group. Between countries, there was a difference in the mean duration of hospitalization and antibiotic treatment, which probably can be explained by broad diversity in local policy [16]. In risk category ‘infection possible,’ costs of health care were found to be (not significantly) higher for PCT-guided decision making. This was an unexpected finding, as duration of hospitalization was expected to be shorter in the PCT-guided decision-making group. Considering the sensitivity analyses excluding SAEs, it was found that duration of hospitalization and total costs were significantly shorter and lower for PCT-guided decision making, compared to standard care. This indicates that neonates in the PCT-guided decision-making group had higher costs and longer hospitalization than neonates in the standard care group due to SAEs. Although exclusion of SAEs reflects the true potential of the intervention on reducing costs and hospitalization, in clinical practice additional days due to prematurity related problems will remain. However, reducing intravenous antibiotic therapy will not only benefit the duration of treatment/hospitalization, but is also important for improving mother and child bonding, reducing the use of monitor facilities, reducing additional procedures as IV-catheters and moreover reducing the possible alterations microbiome that could possibly lead to microbial resistance. For risk category ‘infection unlikely,’ on the other hand, health care costs were significantly lower for PCT-guided decision making. This was an expected result as treatment in this risk category was affected by the intervention. However, sensitivity analyses showed that a higher price of PCT-testing resulted in a nonsignificant difference. Therefore, a significant cost reduction for PCT-guided decision making depends on the price of PCT-testing. The price difference in PCT-tests between laboratories is mainly caused by variation in number of PCT-tests that are performed in a hospital. The more PCT-tests a hospital performs, the lower the costs per test can be.

### Comparison to previous studies

To the best of our knowledge, there have been no cost-effectiveness studies on PCT-guided decision making in children. Several studies have been performed on costs of PCT-guided decision making in adults with sepsis, which found significant health care costs reductions when PCT-guided decision making was applied [8, 9, 11, 12]. One study, however, found no significant difference in health care costs during initial hospitalization in critically ill adults, which is in line with our findings [10]. The subgroup analyses of our study, however, showed a significant reduction in costs for risk category ‘infection unlikely’ when PCT-guided decision making was applied.

A previous study on PCT-guided decision making found that antibiotic use and adverse events related to antibiotic use could be significantly reduced in children aged 1 month to 14 years old with pneumonia [17]. The significant reduction in antibiotics use was in line with our findings, and led to lower costs of antibiotics. Another study in a similar population (children aged 1 month to 18 years) with LRTI also found a significant reduction in duration of antibiotic treatment with PCT-guided decision making [18].

### Strengths and limitations

A major strength of this study is that because of the pragmatic approach of the original study, with a large number of neonates in different countries, the results of this cost-minimization analysis can be directly translated into current clinical practice in different clinical settings within the Netherlands. In addition, it provides an estimation of costs in other high-income countries. Even though there is variation in health care costs within these high-income countries, this study still shows that PCT-guided decision making remains to be cost-effective in the lowest risk category. However, cost estimations for other countries should be interpreted with caution, as the resource prices were calculated using the Dutch price, corrected for PPP for GDP.

One major limitation of our study is that duration of stay at the hospital is possibly biased due to SAEs. The majority of the SAEs resulted in prolonged hospitalization due to prematurity related causes, like feeding intolerance or hyperbilirubinemia [7]. Therefore, duration of hospital stay due to sepsis is overestimated for these patients. However, the sensitivity analysis, using duration of hospitalization excluding SAEs, showed the real potential of PCT-guided decision making.

Furthermore, costs in this study only represent health care costs. Additional societal costs, such as costs from missed work days of the care-givers of the neonates, were not taken into account. Since not all countries provide extensive parental leave, especially for the partner, this could induce additional costs.

### Implications for practice

Applying antibiotic stewardship in neonates suspected with EOS has several practical benefits, besides reducing the duration of antibiotic therapy and diminishing long-term effects on a neonates health. Firstly, there will be less use of cardiorespiratory monitoring, which is particularly interesting in hospitals with limited monitoring capacities. Subsequently, less antibiotic treatment also implicates a reduction in use of personnel hours: from less need to prepare intravenous medication, to less attempts to gain intravenous access to administer antibiotic therapy. Due to the COVID pandemic, the threshold to implement PCT in laboratories is lowered. PCT-testing is now possible on almost all platforms used by the majority of the hospital laboratories. A higher use and availability of PCT-testing will also lower the cost price of procalcitonin testing, resulting in larger cost reductions.

## Conclusion

In conclusion, applying PCT-guided decision making in neonates born after 34 weeks of gestational age, with a low risk of suspected EOS in the first 72 h of life requiring antibiotic therapy, resulted in a nonsignificant difference in total health care costs compared to standard care. A significant cost reduction was found for subgroup ‘infection unlikely.’ However, cost reduction is affected by both the price of PCT-testing and hospitalization due to SAEs.

## Supplementary Information

Additional file 1: Figure S1. Risk classification and duration of antibiotic therapy using normal values of procalcitonin [7].

Additional file 2: Table S1. Overview cost prices in 2015€.

## Data Availability

Not applicable.

## Abbreviations

PCT: Procalcitonin
EOS: Early-onset sepsis
SAE: Serious adverse event
LRTI: Lower respiratory tract infection
CRF: Case record form
NL: Netherlands
CH: Switzerland
CA: Canada
IQR: Interquartile range
