# Supplementary material for: Cost impact of procalcitonin-guided decision making on duration of antibiotic therapy for suspected early-onset sepsis in neonates
Source: Crit Care. 2021 Oct 20;25:367. doi: 10.1186/s13054-021-03789-x (PMC8529813; doi:10.1186/s13054-021-03789-x)
Supplement: Supplementary file 1 — Risk classification and duration of antibiotic therapy using normal values of procalcitonin [7]. [file 13054_2021_3789_MOESM1_ESM.docx]

|  |  | **Duration of antibiotic therapy** |  |
| --- | --- | --- | --- |
| **Risk category** | **Description** | **PCT-guided decision making** | **Standard care** |
| Category 4 | Infection unlikely – low risk, negative culture | At least 24 h, stop after 2 consecutive procalcitonin values within range | 36- 72 h |
| Category 3 | Infection possible – low risk, negative culture |  | 5 – 7 days |
| Category 2 | Infection probable – high risk, negative culture | No PCT-guided treatment 7 – 21 days depending on local policy | |
| Category 1 | Infection proven – high risk, positive culture |  |  |

Figure A1: Risk classification and duration of antibiotic therapy using normal values of procalcitonin [7]
