# Supplementary material for: Cost impact of procalcitonin-guided decision making on duration of antibiotic therapy for suspected early-onset sepsis in neonates
Source: Crit Care. 2021 Oct 20;25:367. doi: 10.1186/s13054-021-03789-x (PMC8529813; doi:10.1186/s13054-021-03789-x)
Supplement: Supplementary file 2 — Overview cost prices in 2015€. [file 13054_2021_3789_MOESM2_ESM.docx]

**Table A1: Overview cost prices in 2015€**

| Item | Unit | Price NL | Price CH | Price CA |
| --- | --- | --- | --- | --- |
| Hospital day generic | Day | €445.70 | €570.50 | €439.00 |
| Hospital day academic | Day | €645.90 | €826.70 | €636.20 |
| Lab CRP | Test | €4.10 | €5.20 | €4.00 |
| Lab PCT | Test | €19.70 | €25.20 | €19.40 |
| Lab PCT min.^a^ | Test | €13.70 | €17.50 | €13.50 |
| Lab PCT max.^b^ | Test | €33.90 | €43.40 | €33.40 |
| Amoxicillin | Pack | €1.40 | €1.80 | €1.40 |
| Penicillin | Pack | €2.30 | €2.90 | €2.20 |
| Gentamycin | Pack | €3.80 | €4.80 | €3.70 |
| Ceftazidim | Pack | €7.10 | €9.10 | €7.00 |
| Physician generic | Hour | €44.40 | €56.80 | €43.70 |
| Nurse generic | Hour | €20.60 | €26.40 | €20.30 |
| Physician academic | Hour | €53.70 | €68.70 | €52.90 |
| Nurse academic | Hour | €18.40 | €23.60 | €18.20 |

NL Netherlands CH Switzerland CA Canada
NA Not Applicable
^a^Lowest price found for PCT test
^b^Highest price found for PCT test
